# Cough Remedies

**Published:** 1877-12

**Authors:** 


Most of the so-called cough remedies should be suppressed, because they contain opium, and beside doing positive injury to the delicate organs of the throat, induce a taste for opium and the destructive habit of opium eating. We doubt whether any cough remedy is of permanent benefit; but, as there are thousands of people who will use them, they should have the best, and in these times the cheapest. The best and cheapest, then, are "Dr. Hall's Troches." They contain no opium, and are very pleasant to the taste, and those who have used them always prefer them to any other. They are put up neatly in boxes of the usual size, and sold at about half the usual price; that is, at 25 cents a box, or five boxes for $1.00. Sent free by mail, on receipt of price, by Hall & Ruckel, 218 Greenwich street, New York.

---

### GOOD EYES.

Life is deprived of most of its joys if sight is imperfect. This fact makes it important that all aids to vision should be so intelligently applied as to accomplish the desired results with safety to the delicate organs involved. A scientific and skillful optician is a very valuable member of society. We have heretofore spoken of Mr. I. Kahn, the head of the New York Optical Institute, 74 Fourth Avenue. He had a case of his beautiful self-adjusting eye-glasses on exhibition at the late fair of the American Institute, for which he received the Society's "medal of excellence"—an honor well deserved. We know that multitudes of persons are wearing spectacles which they have selected for themselves, or which have been supplied by some merchant or watchmaker. Many good eyes have been badly injured or totally ruined by improper glasses. It has greatly surprised us to observe how little most persons seem to care whether the seller of spectacles be a competent optician or an ignoramus. It costs not one penny more to buy a pair of pebbles—and we are of the opinion that clear pebbles are the only lenses fit to place before the eye—of a scientific gentleman like Mr. Kahn, than from the gun-smith or clock-maker, who keeps a few spectacles among his other fancy knick-knacks. Because Mr. Kahn has knowledge and skill, and experience, and judgment, and a high aim and sterling honesty, we constantly avail onrselves of his services, and warmly commend him to our readers and patients.

